# Association Between Gender, Age, and Skeletal Class With Mandibular Condyle Morphology: A Retrospective Study

**DOI:** 10.7759/cureus.49043

**Published:** 2023-11-19

**Authors:** José Carlos López Ramírez, Jairo Mariel Cárdenas, Francisco Javier Gutiérrez Cantú, Marco Felipe Salas Orozco, Carlo Eduardo Medina-Solís, Yolanda Hernández Molinar, Edith Trejo Rivero, Nuria Patiño-Marín

**Affiliations:** 1 School of Stomatology, Autonomous University of San Luis Potosí, San Luis Potosí, MEX; 2 Dentistry, Institute of Health’s Sciences, Autonomous University of the State of Hidalgo, Hidalgo, MEX; 3 School of Stomatology, Clinical Research Laboratory, Autonomous University of San Luis Potosí, San Luis Potosí, MEX

**Keywords:** morphometric analysis, skeletal class, cephalometric analysis, panoramic radiography, mandibular condyle morphology

## Abstract

Objectives: This study aimed to examine the impact of demographic variables on mandibular condyle morphology, a critical factor in orthodontic treatment and maxillofacial surgery. The investigation focuses on the relationship between gender, age, and skeletal class with the morphological dimensions of the condyle, utilizing panoramic radiography as a diagnostic tool.

Methodology: A retrospective analysis was conducted on 150 panoramic radiographs from individuals stratified into six groups according to gender and skeletal class. Skeletal classes were determined using Steiner and McNamara cephalometry. The Kodak Carestream software (Rochester, NY: Carestream Health) was employed to measure condylar height, width, and morphology. Statistical evaluations included ANOVA, correlation assessments, and multivariate binary logistic regression to discern the differences and associations among the variables studied.

Results: The findings revealed notable differences in condylar dimensions between genders across different skeletal classes, with males typically presenting larger condylar dimensions than females. The data also showed a moderate positive correlation between condyle height and width. Round-shaped condyles were the most common form found, with significant gender differences observed in certain skeletal classes. Additionally, logistic regression analysis identified significant associations between gender, age, and condylar width and shape.

Conclusions: The study concludes that demographic factors, such as gender and age, significantly affect mandibular condyle morphology. These factors should be carefully considered in clinical evaluations using panoramic radiography to enhance the precision of diagnoses and the effectiveness of subsequent orthodontic and maxillofacial treatments. The results provide valuable insights for healthcare professionals in regions where more advanced imaging techniques may not be readily available.

## Introduction

The temporomandibular joint (TMJ) is one of the most complex joints in the human body. The mandibular condyle articulates with the glenoid fossa of the temporal bone, and between these structures is the articular disc, a fibrocartilaginous tissue that allows smooth sliding and acts as a cushion between the condyle and the fossa. The mandibular condyle is an essential structure in the temporomandibular joint, allowing mobility and function of the jaw. Under normal conditions, the mandibular condyle should be positioned in a way that allows proper jaw function without interference or pain, maintaining a harmonious relationship with the glenoid fossa and the articular disc. This involves a complex biomechanical interaction that requires a balance between anatomy and function [1].

The position, size, and shape of the mandibular condyle can be determined anatomically and radiographically, using techniques such as panoramic radiography [2], magnetic resonance imaging [3], and computed tomography [2]. The morphology of the condyle can vary depending on individual anatomy, jaw function, genetic factors, environmental factors, age, gender, occlusal load, laterality (differences between the right and left sides of the face), and different types of malocclusions.

Variations in the shape and size of the condyle can influence the position and function of the jaw, which in turn can be associated with sagittal skeletal malocclusions. However, the full understanding of this relationship is not yet well established, especially in specific populations [4]. Sagittal skeletal malocclusions constitute a common dental problem that reflects an alteration in the anteroposterior relationship between the jaws, being classified as class I, II, or III according to the relative position of the maxilla and the mandible. These malocclusions not only affect facial aesthetics but can also have a negative impact on masticatory function and, therefore, the quality of life of affected individuals [5].

The Mexican population presents significant genetic and environmental diversity, which can influence the manifestation and characteristics of both skeletal malocclusions and condylar morphology. Despite the clinical relevance of understanding this relationship, the existing literature regarding the correlation between condylar morphology and sagittal skeletal malocclusions in the Mexican population is limited. This gap in knowledge highlights the need to generate various research studies in this population to improve the understanding of these relationships and their clinical implication [6].

The objective of this study was to associate gender, age, and skeletal class with the morphology of the mandibular condyle.

## Materials and methods

A retrospective study was conducted from 2014 to 2021 at the Master’s Degree in Dental Science with a specialization in the Advanced General Dentistry Program, Autonomous University of San Luis Potosí, San Luis Potosi, Mexico. The study received approval from the Research Ethics Committee of the Autonomous University of San Luis Potosi (#CEI-FE-024-021).

Study population, sample selection, and groups

A total of 150 participants with clinical records met the selection criteria. Consecutive non-probabilistic sampling was utilized. The records were divided into six groups as follows: (1) 25 males with skeletal class I, (2) 25 females with skeletal class I, (3) 25 males with skeletal class II, (4) 25 females with skeletal class II, (5) 25 males with skeletal class III, and (6) 25 females with skeletal class III. The inclusion criteria were as follows: (a) participants of both genders; (b) those with skeletal classes I, II, and III; (c) aged between 14 and 70 years; (d) patients requiring orthodontic treatment; (e) patients with radiological studies; and (f) presence of 28 permanent teeth in the mouth. Exclusion criteria included the following: (a) unobservable radiographs, (b) patients with mixed dentition, and (c) patients with a history of maxillofacial surgery.

Sample size calculation

Sample size was calculated using a mean comparison, based on a prior study, accounting for the differences between skeletal class and condyle morphology [4]. The smallest calculated sample size was 20 participants in each group, with a power of 0.80 and a significance level of 0.05.

Dental panoramic radiographs and image analysis

Radiographs

Panoramic radiographs were acquired at the radiology department of the Faculty of Stomatology, Autonomous University of San Luis Potosí, using the Kodak CS 9300 radiological equipment (Rochester, NY: Carestream Health). Patients were positioned vertically as the device rotated around their heads. A meter ensured the true vertical position, guaranteeing that the radiological image was error-free.

Skeletal Class

To categorize each patient's skeletal class, Steiner and McNamara cephalometry was utilized. Measurements included skeletal class determination. The cephalometric points measured in each cephalometric analysis are described below.

Steiner: The cephalometric analysis included the angle formed by the sella, nasion, and point A (SNA); the angle formed by the sella, nasion, and point B (SNB); angle formed by point A, nasion, and point B (ANB); distance from sella and nasion to point D (SND); segment representing the sella-lambda line (SL segment); distance between the sella and point E (SE segment); angle between the gonion-gnathion line and the sella-nasion line (GO-GN/SN angle); occlusal plane to SN; angle between the upper canine incisal plane and nasion (ICS-NA angle); ICS-NA distance; palatal plane/ICS; angle formed between the lower canine incisal plane and nasion-B (ICI-NB angle); ICI to GO-GN plane; and interincisal angle.

McNamara: The cephalometric analysis included nasion perpendicular to point A, effective mandibular length condilion-gnation, length condilion-point A, maxillo-mandibular difference, anteroinferior facial height, mandibular plane angle, facial axis angle, nasion perpendicular to pogonion. The results were compared with the standard measurements of each technique to determine the skeletal class.

Condylar Morphology

**Figure 1:**
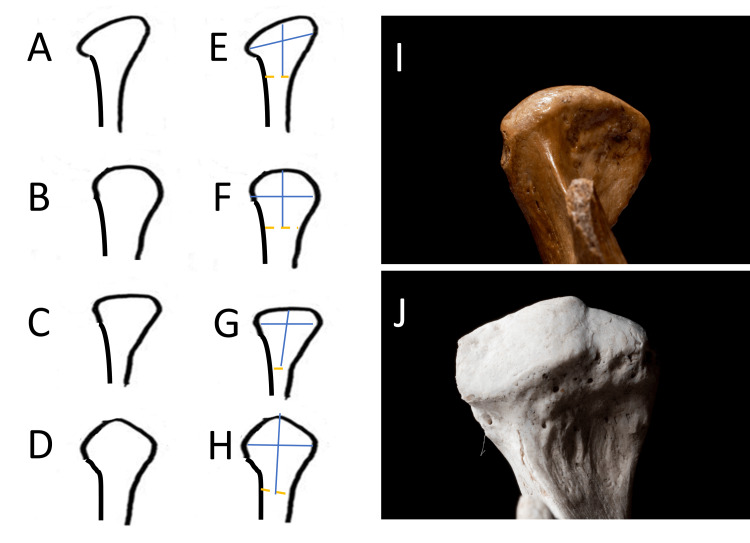
Shapes of the mandibular condyle assessed according to Obërg's classification. (A) Oval shape, (B) round shape, (C) flat shape, (D) inverted V shape, (E-H) measurements of condyle height and width, the dotted line is the base of the condyle, (I) image of dissected mandibular condyle with round shape, (J) image of dissected mandibular condyle with flat shape

The following measurements determined the morphology and dimensions of the mandibular condyle: (1) right mandibular condyle height, (2) right anteroposterior width, (3) left mandibular condyle height, and (4) left anteroposterior width. Measurements were conducted using the Kodak Carestream viewer software (Rochester, NY: Carestream Health), wherein the mandibular condyle's contour was delineated, distinguishing it from the articular fossa and the articular disc space. The height measurement limits were set at the uppermost and medial part of the mandibular condyle and the junction of the condyle head with the neck of the mandibular branch. For width, it was the most posterior and anterior parts of the mandibular condyle. Lastly, the mandibular condyle's morphology was analyzed based on Obërg's classification [7]. The condyles were grouped based on general appearance into the following groups: (1) rounded or slightly convex, (2) mostly flat (straight), (3) ridge-shaped (inverted V-shaped), and (4) pear (Figures 1A-1J).

Prior to initiating the study, the calibration of the examiner was ensured for all variables. The intraclass correlation coefficients and kappa values obtained during this calibration process exceeded 0.80. In the univariate analysis, categorical variables were represented as frequencies and percentages, while continuous variables were depicted as means with their respective standard deviations. The Shapiro-Wilk and Brown-Forsythe tests were employed to ascertain the distribution of the variables. Differences between groups were identified using the Chi-square test and the Mann-Whitney U test. Ultimately, two multivariate binary logistic regression models were constructed to estimate associations, presented as odds ratios with 95% confidence intervals (CIs) [8]. The dependent variables were defined as follows: (i) gender (male and female) for the first model and (ii) age range, categorized as 14-41 and 42-70 years, for the second model. For both models, the independent variables included the height, width, and shape of the mandibular condyle. A variance inflation factor analysis was conducted to detect and mitigate any multicollinearity among the independent variables. The specification error test was employed to confirm the assumption that the logit of the response variable linearly correlates with the independent variables. After establishing the primary effects, interactions were tested, but none demonstrated significance. To evaluate the overall appropriateness of the model, the goodness of fit test was applied. Data analysis was performed using JMP version 9.0 (Cary, NC: SAS Institute) and Stata version 11.0 (College Station, TX: StataCorp LP).

## Results

In total, 150 participants with clinical records met the selection criteria. The records were divided into the following six groups: (1) 25 males with skeletal class I, aged 14-55 years (mean: 31±1.0); (2) 25 females with skeletal class I, aged 14-70 years (mean: 39±1.0); (3) 25 males with skeletal class II, aged 14-69 years (mean: 35±1.0); (4) 25 females with skeletal class II, aged 15-67 years (mean: 37±1.5); (5) 25 males with skeletal class III, aged 15-69 years (mean: 36±1.0); (6) 25 females with skeletal class III, aged 14-51 years (mean: 34±1.1). On each subject's panoramic radiograph, the height, width, and shape of both the left and right condyles were identified. Table 1 displays the height and width of the left and right condyles for all six study groups. When comparing male and female participants across the three skeletal classes, statistically significant differences were found in the height and width of the mandibular condyle (p<0.05). Regarding height, differences were identified in skeletal classes II (average: 1.23 right, p=0.0001; and 1.10 left, p=0.0013) and III (average: 1.32 right, p=0.0204; and 1.21 left, p=0.0090), with higher values observed in males. When analyzing the width of the condyle, differences were noted in skeletal classes I (average: 1.35 right, p=0.001; and 1.30 left, p=0.001) and II (average: 1.13 right, p=0.001; and 1.06 left, p=0.0109), with lower values found in skeletal class II males. Upon correlating height with condylar width across all groups, a moderate positive correlation was observed (Rho=0.4709, p=0.0001).

**Table 1:** Height and width of the mandibular condyle in the study groups. The statistical test used was Mann-Whitney U test and n=25 X-rays/group.

Condyle		Height (mm) mean±SD		Width (mm) mean±SD	
Male (n=75)		Right	Left	Right	Left
Class I		1.08±0.1	1.06±0.2	1.35±0.2	1.30±0.2
Class II		1.23±0.1	1.10±0.1	1.13±0.2	1.06±0.1
Class III		1.32±0.2	1.21±0.2	1.26±0.1	1.18±0.1
Female (n=75)		Right	Left	Right	Left
Class I		1.08±0.1	1.32±0.2	0.91±0.1	1.15±1.2
Class II		0.96±0.1	0.96±0.9	0.96±0.1	0.95±0.1
Class III		1.17±0.2	1.06±0.1	1.24±0.2	1.19±0.2
Comparison: male vs female (p-value)	Class I	0.7923	0.8018	0.0001	0.0001
	Class II	0.0001	0.0013	0.0001	0.0109
	Class III	0.0204	0.009	0.7653	0.1641

The shape of the condyle in the study groups is identified in Table 2. The most common shape in all groups was round (n=106, 35%). When comparing male and female genders with the three skeletal classes, statistically significant differences were found in the shapes of the left condyle of skeletal class II and III (p<0.05), observing high frequencies of the round shape in the male gender with skeletal class III (n=14, 28%) and in the pear shape (n=10, 20%) in women with skeletal class II.

**Table 2:** Shape of the condyle in the study groups. Rt: right; Lt: left; Inv. V: inverted V A chi-square statistical test was used to assess the significance of differences observed across various categories.

Shape		Round		Pear		Flat		Inv. V		Total (n)
Side		Right	Left	Right	Left	Right	Left	Right	Left	
Male (n=75)		Frequency (%)	Frequency (%)	Frequency (%)	Frequency (%)	Frequency (%)	Frequency (%)	Frequency (%)	Frequency (%)	
Class I		11 (22)	8 (16)	1 (2)	6 (12)	8 (16)	5 (10)	5 (10)	6 (12)	50
Class II		10 (20)	10 (20)	7 (14)	7 (14)	4 (8)	6 (12)	4 (8)	2 (4)	50
Class III		9 (18)	14 (28)	6 (12)	7 (14)	3 (6)	1 (2)	7(14)	3 (6)	50
Total		30 (20)	32 (21)	14 (10)	20 (14)	15 (10)	12 (8)	16(11)	11 (6)	150
Female (n=75)		Right	Left	Right	Left	Right	Left	Right	Left	Total
Class I		5 (10)	5 (10)	4 (8)	9 (18)	8 (16)	3 (6)	8 (16)	8 (16)	50
Class II		10 (20)	7 (14)	4 (8)	10 (20)	5 (10)	1 (2)	6 (12)	7 (14)	50
Class III		10 (20)	7 (14)	3 (6)	4 (8)	9 (18)	6 (12)	3 (6)	8 (16)	50
Total		25 (17)	19 (13)	11 (6)	23 (15)	22 (15)	10 (7)	17 (11)	23 (16)	150
Total (n=300)		55 (18)	51 (17)	25 (8)	43 (14)	37 (12)	22 (7)	33 (11)	34 (13)	300
Comparison: male vs female (p-values)	Class type	Right	Left	-	-	-	-	-	-	-
	Class I	0.1768	0.553	-	-	-	-	-	-	-
	Class II	0.719	0.0467	-	-	-	-	-	-	-
	Class III	0.1188	0.023	-	-	-	-	-	-	-

In Table 3, the combinations of left and right condyle shapes most common in each study subject are observed. Statistically significant differences were identified (p=0.0001) in class I of the female gender and in class II of both genders.

**Table 3:** Combinations of left and right condyle shapes most frequent in each study subject. A chi-square statistical test was used to assess the significance of differences observed across various categories. Each skeletal class consisted of a sample size of 50.

Male (75)		Frequency (%)	p-Value	Female (75)	Frequency (%)	p-Value
Class I	(1) Round/flat	7 (14)	0.0925	(1) Pear and pear	8 (16)	0.0001
				(2) Pear and inverted V		
Class II	(1) Round and round	11 (22)	0.0001	(1) Round and round	8 (16)	0.0001
				(2) Inverted V and inverted V		
Class III	(1) Round and round	6 (12)	0.4537	(1) Round and flat	8 (16)	0.1288
	(2) Round and flat			(2) Round and inverted V		
	(3) Round and inverted V					

Multivariate binary logistic regression analysis for males and females and age range is identified in Table 4. The two variables were associated (p<0.05) with the width (male and female OR=8.0; and age range OR=9.0) and with the shape of the condyle (male and female OR=4.0; and age range OR=6.0).

**Table 4:** Multivariate binary logistic regression analysis for male, female, and age range.

First model dependent variable: male and female			
Independent variables	OR	95% CI	p-Value
Height	3	0.48-18.94	0.2232
Width	8	1.16-1.22	0.0365
Shape	4	0.3-0.82	0.0422
Second model dependent variable: age range (14-41 and 42-70)			
Independent variables	OR	95% CI	p-Value
Height	2	0.3-19.0	0.4333
Width	9	1.7-1.99	0.0195
Shape	6	0.6-1.2	0.0005

## Discussion

The aim of this study was to determine the association between gender, age, and skeletal class with condylar morphology. The study was conducted using panoramic radiography to assess condylar shape and determine skeletal malocclusion. Panoramic radiography was chosen due to its numerous advantages, especially in emerging or developing countries like Mexico. For instance, panoramic radiography is a cost-effective imaging technique compared to CBCT, it uses lower radiation doses than CBCT. It is more accessible since CBCT requires more specialized equipment and knowledge. It is useful for general dental and orthodontic evaluations. It is also less prone to image distortions from movement than CBCT [9].

In this study, when comparing male and female genders across the three skeletal classes, statistically significant differences were found in the height and width of the mandibular condyle (p<0.05). Higher values in height were observed in skeletal class II and III in males. These results seem to align with previous studies. Tariq and Jan found that in general, mandibular condyles tend to be larger in individuals with skeletal class III and smaller in individuals with skeletal class II. They also found that the mandibular condyle in males tends to have larger morphometric parameters compared to females [10]. Saccucci et al. found that in a Caucasian population, skeletal class III exhibited a larger condylar volume compared to skeletal class I and II. Likewise, the volume and surface area of the mandibular condyle were larger in males than in females [11]. However, the study by Mohsen et al. did not report significant differences between the morphometric parameters of the mandibular condyle and the sagittal or vertical skeletal class [12]. Skeletal class III malocclusion is associated with a greater height of the mandibular condyle because patients with skeletal class III have a horizontal growth pattern in their jaw [13]. This is due to the fact that class III malocclusion often represents mandibular prognathism, which is primarily of genetic origin [14]. Additionally, skeletal class III can sometimes represent progressive condylar hyperplasias, which are characterized by excessive growth of the mandibular condyle, which is generally unilateral, also causing facial asymmetry [15].

According to our results, we also observed greater width of the mandibular condyle in subjects with skeletal class II. However, based on the literature review we conducted, this type of relationship has not been previously reported, and some studies even report contradictory results. For instance, Mohsen et al. did not find significant differences between the width of the mandibular condyle and the sagittal skeletal classes [12]. Whereas Alhammadi et al. reported that subjects with skeletal type II malocclusion had a narrower condylar width [16]. Hasebe et al. also reported that patients with skeletal class II had mandibular condyles of narrower width [17]. Although it has not been well studied, the reason why the condyle has a greater width in subjects with class II malocclusion may be due to the adaptive growth phenomenon. In skeletal class II, especially in cases where there is mandibular retrognathism (the jaw is positioned backward), the condyle might adapt and remodel to compensate for the deficient mandibular growth [18]. Additionally, the position and function of the temporomandibular joint (TMJ) may be altered, leading to increased stress and adaptive changes in the condylar region [19].

Our data indicate differences in the width of the mandibular condyle between males and females with skeletal class type I. This result may be due to the inherent differences in anatomy and morphology between males and females, and different biomechanical factors. For instance, Lewis et al. reported that the translational movements of the mandibular condyle during opening and closing movements were greater in males (approximately 15-20 mm) than in females (approximately 12-17 mm); both in linear and curvilinear movements. This is explained by the authors' findings that males had a larger jaw compared to females. Likewise, the mandibular rotational movement during opening was significantly greater in males compared to females [20]. Chen et al. reported that the maturation of chondrocytes in the mandibular condyle is affected in female mice and not in male mice when there are changes in occlusal pressure [21]. Jiao et al. reported that the development of the condylar cartilage and subchondral bone occurs at earlier stages of development (before four months) in female rats compared to males. As a result, this leads to a larger condyle at an earlier age in female rats [22].

Our results indicate that the most common condyle shape in all study groups was the round shape of the right condyle (n=55, 18%). In the male gender, this condylar shape predominated on the left side (n=32, 21%), and in the female gender, it predominated on the right side (n=25, 17%). Yalcin and Ararat conducted a study in 2019 in which they evaluated the morphology of the mandibular condyles in a Turkish population. The authors found a significant difference in the shape of the right condyle between males and females. In females, the shape of the right condyle was rounded, while males presented a flattened right condyle. Differences in our results may be due to the methodology used; the aforementioned authors used a classification of condylar morphology different from ours. Moreover, the authors also associate these differences with genetic factors, such as racial morphological differences, and the presence of functional factors like the degree of edentulism in the patients evaluated [23]. Singh et al. conducted a study in 2020 in which they analyzed panoramic radiographs of 350 patients. The study's results reported that the predominant morphology of the mandibular condyle was the rounded shape with a prevalence of 62%. This result is consistent with our finding that the most common condylar shape was the rounded form at 35%. However, unlike our results, the authors report that 80% of the patients had the same rounded condylar shape on both sides. Similarly, the authors reported that tooth loss on both sides of the arch significantly affects the shape of the mandibular condyle [24]. These results are consistent with the findings of Richards [25]. Another study in 2020, which was conducted on a sample of 450 digital panoramic radiographs of Iraqi patients, also reported that the most common shape of the mandibular condyle was the rounded (oval) shape, which was present in a similar percentage in both males and females (55%). This result aligns with our study's findings, even though the method of classifying the condylar morphology was different from the one we used. Just like in this study, the authors reported a significant difference in the condyle shape between the right and left sides and between the genders of the patients [26].

Regarding the skeletal class, in this study, the most frequent shape of the condyle was the rounded shape on the left side in the male gender with class III (14, 18%) and the rounded shape on the right side in class II, III, and pear-shaped on the left side in class II (10, 20%) in the female gender. However, Merigue et al. found no association between the shape of the condyle and the type of malocclusion present in the patients. The authors reported that the most frequent shape of the mandibular condyle in the Brazilian population was convex [27]. Mohsen et al. also found no association between the coronal or sagittal shape of the condyle and the sagittal and vertical skeletal malocclusion patterns of a Chinese population [12]. Sreenivasagan et al. reported that the oval-shaped mandibular condyle was the most common, followed by the bird's beak shape in the three types of malocclusions (I, II, and III) present in an Indian population [28]. Furthermore, a large number of studies report that the most common shape of the mandibular condyle is the rounded or oval shape, and there does not seem to be a relationship between this and the horizontal skeletal malocclusion pattern. In a recent systematic review, the results indicated that there is a clear relationship between morphological changes in the mandibular condyle and different types of malocclusions. Such morphological changes in the mandibular condyle are more common in sagittal malocclusions but more severe in vertical malocclusions [29].

Finally, our results found an association between gender and age with the shape and width of the mandibular condyle. This finding aligns with previous studies; for instance, Karlo et al. reported that with increasing age, the size of the condyles tended to grow, which in turn caused a reduction in the anteversion angles and modified the condyle shape from round to oval [30]. Yalcin and Ararat found that mandibular condyles had a convex shape in the age ranges of 18-29 years and 30-59 years, while in the age range of over 60 years, patients displayed an angled condylar shape. Additionally, they also found an association between gender and the shape of the right condyle. The right condyle in females tended to be more rounded compared to males [23]. Tassoker et al. reported that subjects older than 56 years tend to have mandibular condyles with a flattened or angled shape [31].

The present study, with its retrospective design, faces inherent limitations associated with this methodology, as retrospective data collection may limit our ability to infer causal relationships and exert control over all relevant variables, in addition to the possibility that relevant information may not have been collected at the time of the original creation of clinical records. Despite having used a sample size calculation based on previous research, we must acknowledge that the sample size used may not make it feasible for the results to be extrapolated to the entire Mexican population.

## Conclusions

In this study, an association between age and gender with the shape of the mandibular condyle was identified. The findings suggest that considering these demographic variables can enhance clinical accuracy in predicting these condylar features. This information can be used in the planning of orthodontic treatments and maxillofacial surgeries when more precise evaluation methods, such as tomography or magnetic resonance imaging, are not readily available.
